# Shoulder Motion Following Combined Glenoid Anteversion Osteotomy Compared with Soft Tissue Rebalancing Alone for Brachial Plexus Birth Injury

**DOI:** 10.2106/JBJS.OA.25.00274

**Published:** 2025-12-10

**Authors:** Deeptiman James, Alison Anthony, Howard Clarke, Kristen Davidge, Sevan Hopyan

**Affiliations:** 1 Division of Orthopaedic Surgery, The Hospital for Sick Children, Toronto, Canada; 2 Current Address: Department of Orthopaedics, Shri Balaji Institute of Medical Sciences, Raipur, India; 3 Rehabilitation Services, The Hospital for Sick Children, Toronto, Canada; 4 Division of Plastic & Reconstructive Surgery, Hospital for Sick Children, Toronto, Canada; 5 Division of Plastic, Reconstructive, & Aesthetic Surgery, Department of Surgery, University of Toronto, Toronto, Canada; 6 Division of Orthopaedic Surgery, Department of Surgery, University of Toronto, Toronto, Canada

## Abstract

**Background:**

Muscle rebalancing improves shoulder internal rotation contracture due to brachial plexus birth injury but is less effective for correcting marked glenohumeral dysplasia. For severe cases, combining glenoid anteversion osteotomy (GAO) with subscapularis lengthening and tendon transfers is an alternative to external rotation osteotomy of the humerus. We asked how the addition of glenoid osteotomy affects shoulder motion.

**Methods:**

We defined 2 groups who underwent very similar procedures with the exception of GAO: GAO group—combined GAO, subscapularis slide, and tendon transfers for severe glenohumeral dysplasia, and non-GAO group—subscapularis slide and tendon transfers without GAO for cases of milder dysplasia. We compared active and passive rotation, Active Movement Scale (AMS) and Mallet scores.

**Results:**

We compared 86 children in the GAO group with 74 children in the non-GAO group with median follow-ups of 58 (IQR1-3:22-101) and 46 (IQR1-3: 24-72) months, respectively. Preoperatively, the children in the GAO group were older (median 79 (range 14-210) months vs. 34 (range 6-204) months) and exhibited a relatively severe distribution of glenohumeral dysplasia than those in the non-GAO group, as expected. The extent of active external rotation (ER) in adduction improved postoperatively in the GAO group by 65° (p < 0.05), and in the non- GAO group by 84° (p < 0.05). Despite loss of the mean end range of internal rotation by 31° and 27°, the total arc of rotation increased by 34° and 57° in the GAO and Non-GAO groups, respectively. At final follow-up, active ER at 90° abduction (p = 0.14), passive ER (p = 0.17), total arc of rotation (p = 0.11), AMS ER (p = 0.45), Mallet global ER (p = 0.9), and Mallet composite (p = 0.9) scores were similar between the groups, irrespective of the glenoid osteotomy.

**Conclusion:**

The 2 approaches compared here resulted in similar functional outcomes despite different initial severities of glenohumeral dysplasia. Addition of GAO for severe cases does not obviate improved motion.

**Level of Evidence:**

Level III. See Instructions for Authors for a complete description of levels of evidence.

## Introduction

### Background

The normal progression of glenoid version and glenohumeral orientation arises during scapular development and requires periarticular muscle balance^1,2^. Development of the bipolar ossification center (between the coracoid process and the superior aspect of the glenoid) and the subcoracoid and glenoid rim ossification centers determine glenoid version and concavity of the glenoid articular surface, respectively^3^. Following brachial plexus birth injury, muscle wasting, glenoid hypoplasia, and retroversion can be detected in the first few months of infancy and contribute to posterior glenohumeral instability^4,5^.

To address internal rotation contracture that is functionally limiting, distinct procedures are performed depending on the degree of glenohumeral dysplasia. Soft tissue rebalancing procedures including subscapularis release and tendon transfers, sometimes combined with joint reduction and posterior capsulorrhaphy, are effective in cases of mild-to-moderate glenohumeral dysplasia. However, soft tissue procedures alone are less reliable at restoring external rotation in cases of severe glenohumeral dysplasia (Water type IV and V), especially in older children with some reports suggesting inferior results over the age of 6 years^6-8^. These reports are instructive, but we take them with some caution as the soft tissue procedures described are somewhat varied.

Traditionally, humeral external rotation osteotomy is reserved for severe glenohumeral dysplasia to place the hand in a more functional position^1,9^. However, that procedure is not intended to address posterior humeral subluxation or to directly alter the balance of external vs. internal rotation power. An alternative approach for severe dysplasia involves soft tissue rebalancing in combination with joint reduction and glenoid anteversion osteotomy (GAO) to improve the deformity closer to its center of rotational angulation^10,11^. In principle, that approach adds passive stability and realigns the fulcrum to facilitate the efficacy of transferred tendons.

### Rationale

The joint-level nature of GAO raises the concern that it potentially stiffens the glenohumeral joint, thereby diminishing the efficacy of soft tissue rebalancing. To address that possibility, we compared the functional outcomes of soft tissue rebalancing with and without GAO. By design, the 2 groups represent different distributions of preoperative severity. We felt this difference would be acceptable for testing the relative merits of GAO, in part because the greater preoperative severity should tend to understate, rather than overstate, any potential advantage of GAO.

## Methods (Detailed in Extended Methods)

### Study Design and Setting

A retrospective cohort study was designed to compare shoulder function after subscapularis slide and tendon transfers with or without combined GAO in children with internal rotation contracture due to brachial plexus birth injury.

### Participants

Participants exhibiting moderate-to-severe glenohumeral dysplasia (Waters III-V) underwent GAO combined with subscapularis slide and latissimus dorsi and teres major tendon transfers and were designated as the “GAO group.” Those with mild-to-moderate glenohumeral dysplasia (Waters I to III) who underwent subscapularis slide and latissimus dorsi and teres major tendon transfers alone were categorized as the “non-GAO group.”

### Surgical Technique

Combined soft tissue rebalancing and GAO with tendon transfers as it was used here was described previously^12^. Differences for the non-GAO group were that (1) only the longitudinal posterior humeral limb of the incision was used, (2) the deltoid origin on the scapular spine was left intact, and (3) the joint capsule was not incised.

### Aftercare

For both groups, casts were removed at 4 weeks and 5 weeks postoperatively, respectively. Participants were then immediately treated by a physiotherapist and learned progressive range of motion and active motion exercises that were continued for a 6-month period at least.

### Outcome Measures

The principal variable we examined was change in the active range of total shoulder motion (not glenohumeral vs. scapulohumeral contributions). Based on these parameters, Active Movement Scale and Mallet Scores were derived.

### Statistical Analysis

The Independent Samples Student *t*-test was used to analyze continuous variables between the groups, and Wilcoxon rank, Kruskal-Wallis, and Mann-Whitney U tests were used to analyze ordinal or categorical variables and skewed data sets.

## Results

### Baseline Parameters

Eighty-six children in the GAO group and 74 children in the non-GAO group had median follow-up of 55 and 41 months, respectively. Consistent with study design differences in dysplasia severity among the 2 groups, the median age at surgery was 79 months in the GAO group and 34 months in the non-GAO group. The gender distributions, laterality, Narakas types, and incidence of previous brachial plexus nerve grafting were similar among the 2 groups (Table I).

**TABLE I:** Baseline Parameters of the Comparison Groups

	GAO Group (n = 86)	Non-GAO Group (n = 74)	p
Age at surgery (in months)			< 0.001
Mean*	93	55	
Median (IQR1; IQR3) [Range]	*79* (47.7; 142) [14 – 210]	*34* (11; 75) [6 – 204]	
Gender*			0.79
Male	40	36	
Female	46	38	
Follow up (in months)			0.13
Mean*	63	52	
Median (IQR1; IQR3) [range]	*58* (22; 101) [6-177]	*46* (24; 72) [6-191]	
Laterality*			0.69
Right	52	47	
Left	34	27	
Narakas type†			0.64
I	31	19	
II	44	52	
III	7	0	
IV	4	3	
Brachial plexus nerve graft*			0.90
Yes	45	38	
No	41	36	

Statistical tools:

* Independent samples *t*-test.

† Wilcoxon rank sum test (for ordinal variables).

### Change in Glenoid Version in the GAO Group

**Fig. 1:**
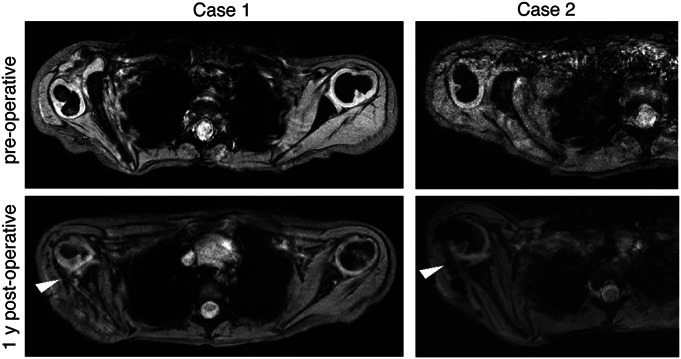
Axial magnetic resonance images of Waters V (Case 1) and Waters IV (Case 2) glenohumeral dysplasia cases that underwent glenoid anteversion osteotomy and soft tissue rebalancing. Transferred latissimus dorsi tendons with signal artefacts due to nonabsorbable sutures are marked by white arrowheads.

In the GAO Group, preoperative MRI for assessment of glenoid version was available for 74 of 86 cases and post-GAO images were available for 72 of 86 cases. The mean preoperative glenoid retroversion following GAO improved from −32.6° (SD 13.5) to −7.5° (SD 13.1, p < 0.05) (Fig. 1, Fig. S1). Following GAO, the distribution of glenohumeral dysplasia severity diminished from predominantly Waters grades III-V to predominantly grades I and II in 59 of 72 children (82%; p < 0.01) (Table S1). Residual moderate-to-severe glenohumeral dysplasia persisted in 13 of 72 children (18%) (6 each with types III and IV and 1 with type V). Quality of the subchondral glenoid fragment was evaluated subjectively on axial T1 images. No cases exhibited evidence of collapse or osteonecrosis either at 1-year or 5-year follow-up.

### External Rotation

**Fig. 2:**
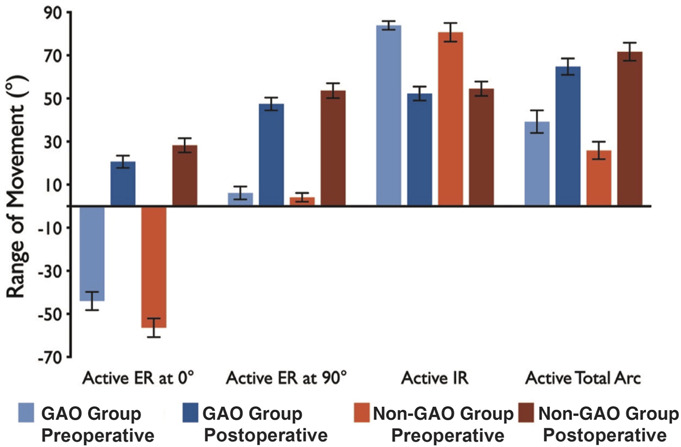
Changes in the active ER (at 0° and 90° of shoulder abduction), IR, and total arc of shoulder rotation in both groups. Bar heights represent means, and error bars represent 1 standard error of the mean above and below the mean. ER = external rotation, and IR = internal rotation.

**Fig. 3:**
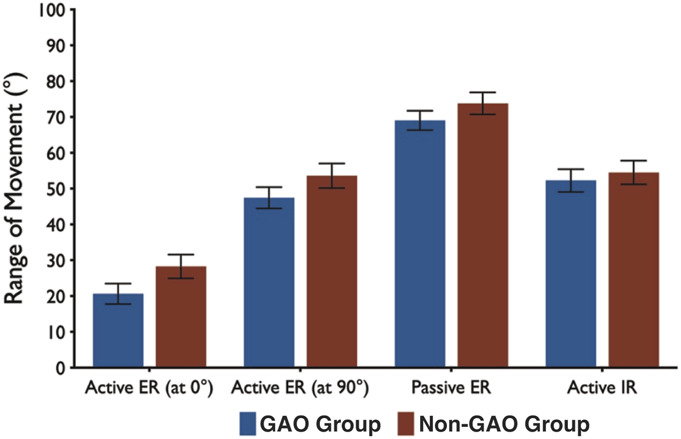
Similar ranges of movements were noted at the final follow-up in both groups. Bar heights represent means, and error bars represent 1 standard error of the mean above and below the mean.

Postoperatively, at the final follow-up, the end range of active ER in adduction had improved by 65° (p < 0.05) and 84.2° (p < 0.05) in the GAO and non-GAO groups, respectively. Similarly, active ER in 90° abduction improved by 46° (p < 0.05) and 50° (p < 0.05) in the corresponding groups (Table II, Fig. 2). At the final follow-up, active and passive ER had improved by a comparable magnitude in both groups irrespective of GAO. Final active ER in adduction was greater in the non-GAO group by 7° (Table S2, Fig. 3).

**TABLE II:** Changes in Shoulder Motion in Each Group

	Preoperative	Final	Difference	p
GAO Group				
Mean active ER (0° shoulder abduction)	−44° (SD: 38.1)	21° (SD: 25)	65	<0.05*
Mean active ER (90° shoulder abduction)	2° (SD: 14)	48° (SD: 28)	46	<0.05*
Mean passive ER	Not available	71° (SD: 23)		
Mean active IR (0° shoulder abduction)	84° (SD: 18)	53° (SD: 27)	31	<0.05*
Active total arc of rotation	40° (SD:46)	74° (SD:34)	34	<0.05*
AMS ER (median) (IQR1; IQR3)	2 (0; 2)	6 (3; 7)	5.7 (Z = −6.5)	<0.05†
Mallet Global ER (median)(IQR1; IQR3)	2 (2; 2)	4 (3; 4)	Z = −9.7	<0.01‡
Mallet Hand-to-mouth	2 (2; 2)	3 (3; 4)	Z = −8.0	<0.01‡
Mallet Hand-to-back	2 (2; 2)	4 (3; 4)	Z = −7.3	<0.01‡
Mallet Composite	12 (11; 13)	16 (14; 17)	4	<0.01‡

Non-GAO Group				
Mean active ER (0° abd)	−56° (SD: 35.6)	28.2° (SD: 28)	84.2	<0.05*
Mean active ER (90° abd)	6° (SD: 12.6)	56° (SD: 29.2)	50	<0.05*
Mean passive ER	Not available	76° (SD: 22)		
Mean active IR	81°(SD: 34.5)	54.5° (SD: 25.8)	26.5	<0.05*
Active total arc of rotation	25° (SD:32.4)	83° (SD: 35.9)	58	<0.05*
AMS ER (median) (IQR1; IQR3)	2 (0; 2)	6 (3; 7)	6.7 (Z = −6.4)	<0.05†
Mallet Global ER	2 (2; 2)	4 (3; 4)	Z = −6.2	<0.01‡
Mallet Hand-to-mouth	2 (2; 2)	3 (3; 4)	Z = −5.8	<0.01‡
Mallet Hand-to-back	2 (2; 2)	3 (3; 4)	Z = −5.6	<0.01‡
Mallet Composite	12 (11; 13)	16 (15; 17)	4	<0.01‡

AMS = Active Movement Scale, ER = external rotation, and Z = based on positive ranks.

Rotation always measured starting from neutral with forearm pointing forward. For ER, positive value indicates rotation away from the body whereas negative value indicates rotation toward the belly. For IR, positive value indicates toward the belly.

Statistical tools:

* Independent samples *t*-test.

† Kruskal-Wallis H test.

‡ Mann-Whitney U test.

Paired preoperative and final AMS scores for shoulder ER were compared for 59 of 86 children in the GAO group and 61 of 74 children in the non-GAO group. The median preoperative AMS score for ER was 2 (IQR1: 0; IQR3:2) and improved to 6 (IQR1:3; IQR3:6, p < 0.05) at the final follow-up in both groups (Table II, Fig. S2). Global ER and hand-to-mouth components of the Mallet scale were analyzed preoperatively for 63 of 86 children postoperatively in 61 of 86 children in the GAO group. In the non-GAO group, 27 of 74 had preoperative scores and 39 of 74 had final scores, in part because of the relatively large proportion of very young children in the non-GAO group for whom Mallet assessment was challenging. With the available data, statistically significant improvements were noted in both Mallet components for both groups (p < 0.01, Mann-Whitney *U* test) (Table II). At final follow-up, AMS ER (p = 0.45) and Mallet global ER (p = 0.9), hand-to-mouth (p = 0.8) and composite scores (p = 0.8) were similar in both groups, irrespective of GAO (Table S2).

### Internal Rotation and Total Arc of Rotation

The end range of active IR was diminished postoperatively by 31° in the GAO group and 26.5° in the non-GAO group. Considering the gain in ER, the active total arc of rotation (TAR) of the affected shoulder improved by 34° and 58° in the GAO group and the non-GAO group, respectively (p < 0.05, Table II, Table S2). At the final follow-up, the 2 groups exhibited a similar TAR range (A: 74°, SD 34; B: 83°, SD 36) (Table S2, Fig. 2).

### Complications

Loss of hand-on-belly and midline ability due to iatrogenic ER contracture was encountered in 7 of 86 children in the GAO group. To address this problem, 2 patients underwent release of posterior scar between the transferred tendons and the underlying tissues, 2 of those patients underwent proximal humeral internal rotation osteotomy, and 3 patients responded to stretching, botox injections, and serial casting. In the non-GAO group, 2 of 74 patients experienced this problem and both underwent humeral internal rotation osteotomy.

Internal rotation contracture persisted unacceptably according to the patient/family and the multidisciplinary team in 5 of 86 children in the GAO group. One patient underwent revision of subscapularis slide, tendon transfers, and GAO to improve ER and another underwent revision of GAO and retensioning of transferred tendons. Both patients who underwent redo GAO achieved good ER at the final follow-up (AMS score 6). The remaining 3 children underwent revision tendon transfers using lower trapezius transfer augmented by allograft tendon extenders. Their AMS ER scores improved at the final follow-up in 2 of those patients: (1) 2 to 6, (2) 2 to 5, and (3) 2 to 2. In 1 patient, ER recovery was complicated by an unrelated ipsilateral proximal humerus fracture. They ultimately achieved active ER of 40° and an AMS ER score of 6 at the final follow-up.

In the non-GAO group, internal rotation contracture persisted in 9 of 74 children. Three patients underwent tendon transfer revision and lower trapezius transfer with allograft tendon augmentation as well as Botox injection to pec major and subscapularis. One patient underwent revision tendon transfer combined with GAO and was therefore included in the GAO group after revision surgery. One child underwent imbrication of the transferred tendons at a different center. The AMS ER scores in children who underwent reoperation improved significantly to AMS 6 or 7.

Surgical site infection occurred in 1 patient in the non-GAO group and none in the GAO group. One patient underwent repeat open reduction of an inferiorly dislocated glenohumeral joint and another in the GAO group suffered a fracture of the inferior part of osteotomy site, but neither event obviously affected the functional outcome. Chronic symptomatic GH instability with gross restriction of shoulder function was present in 1 patient in the GAO group and 2 in the non-GAO group. The glenohumeral joint was fused in 2 of these patients.

## Discussion

### Progression of Glenohumeral Dysplasia

Glenohumeral dysplasia often worsens, and remodeling potential diminishes with age^13^. Appearance of the subcoracoid secondary ossification center by about the age of 8 years corresponds to substantive reduction of developmental glenoid remodeling^3^. The degree of glenohumeral dysplasia in brachial plexus birth injury correlates with atrophy of the rotator cuff and deltoid muscles^5^, incomplete and prolonged palsy, imbalance between the external and internal rotators, and increasing age^4^. Moderate glenohumeral dysplasia can be improved (Waters grade 3-2) by release of contracture and tendon transfers^14^, especially when undertaken under the age of 2 years but less so in older children^6^. Since advanced glenohumeral dysplasia impedes the success of soft tissue procedures^8^ and persists even in some young children despite a release of contracture and tendon transfers^15^, alternative procedures have been devised.

### Osteotomies for Severe Glenohumeral Dysplasia

Rotational osteotomy of the humerus is a longstanding approach to place the hand in a more functionally useful position more directly in front of the patient^9,16-19^. *External* rotation osteotomy of the humerus without soft tissue rebalancing may achieve this goal or at least improve the appearance of the affected limb^9,16,17^. A potential downside is worsening of posterior instability of the humeral head^20^. A less common approach is humeral *internal* rotation osteotomy in conjunction with open reduction to achieve a more congruent glenohumeral joint^20-22^. Some of these reports suggest that humeral rotational osteotomy is most likely to be beneficial when glenohumeral dysplasia is mild and when it is performed at a relatively young age. In practice, humeral osteotomy is often recommended for more severe dysplasia (Waters grade IV +) in older children in whom the deformity will persist^9^.

### Combined Glenoid Anteversion Osteotomy and Soft Tissue Rebalancing as an Alternative to Humeral Osteotomy

The conceptual appeal of glenoid osteotomy is to transform dysplasia from severe to mild for the purpose of enabling efficacy of soft tissue rebalancing. The early outcomes reported previously^10,11^, and midterm outcomes reported here show that improved joint reduction and glenoid version are achieved and maintained by a combined approach. Improvements in active and passive shoulder external rotation here were comparable with those of other reports of soft tissue rebalancing^13,14,18,23^.

In our opinion, the ideal balance of active rotation would be from the palm-on-belly position in internal rotation to just beyond neutral in external rotation to facilitate activities in the midline, in front of the torso, and ability to reach the face and head. The challenge of achieving that ideal is reflected in similar rates of unsatisfactory outcomes requiring reoperation in both groups here and are comparable with those reported elsewhere^13,18,19,23,24^.

## Limitations and Future Directions

The retrospective, single center setting of this study is associated with inherent selection and ascertainment biases. Patient-reported assessment of function and quality of life, and race and ethnicity data are also lacking. On the other hand, the relatively large cohort sizes, standardized perioperative protocols, and midterm follow-up are strengths of this comparison. Since the 2 groups have different indications based on dysplasia severity, this comparison is not intended to assist decision making. Rather, it serves as an assessment of the safety and function of glenoid osteotomy. A comparison between glenoid and humeral osteotomy would have more real-world implications for decision making since their indications would be similar.

## Conclusions

Addition of glenoid anteversion osteotomy did not negatively affect the motion outcome or the complication profile of soft tissue rebalancing despite the greater initial severity of glenohumeral dysplasia. We speculate that intraoperative steps taken to avoid iatrogenic external rotation contracture (as described in the Extended Methods) help to optimize the outcome. The glenoid osteotomy approach is a viable alternative to humeral osteotomy for severe glenohumeral dysplasia.

## Appendix

Supporting material provided by the authors is posted with the online version of this article as a data supplement at jbjs.org (http://links.lww.com/JBJSOA/B35). This content was not copyedited or verified by JBJS.

## References

[1] Eichinger JK Galvin JW Grassbaugh JA Parada SA Li X. Glenoid dysplasia: pathophysiology, diagnosis, and management. J Bone Jt Surg Am. 2016;98(11):958-68. 10.2106/JBJS.15.00916 27252441

[2] Mintzer CM Waters PM Brown DJ. Glenoid version in children. J Pediatr Orthop. 1996;16(5):563-6. 8865036 10.1097/00004694-199609000-00002

[3] Kothary S Rosenberg ZS Poncinelli LL Kwong S. Skeletal development of the glenoid and glenoid-coracoid interface in the pediatric population: MRI features. Skeletal Radiol. 2014;43(9):1281-8. 24986651 10.1007/s00256-014-1936-0

[4] Iorio M Menashe SJ Iyer RS Lewis SP Steinman S Whitlock KB Tse RW. Glenohumeral dysplasia following neonatal brachial plexus palsy: presentation and predictive features during infancy. J Hand Surg Am. 2015;40(12):2345-51 e1. 26541441 10.1016/j.jhsa.2015.08.029

[5] Van Gelein Vitringa VM Jaspers R Mullender M Ouwerkerk WJ Van Der Sluijs JA. Early effects of muscle atrophy on shoulder joint development in infants with unilateral birth brachial plexus injury. Dev Med Child Neurol. 2011;53(2):173-8. 20846159 10.1111/j.1469-8749.2010.03783.x

[6] Van Heest A Glisson C Ma H. Glenohumeral dysplasia changes after tendon transfer surgery in children with birth brachial plexus injuries. J Pediatr Orthop. 2010;30(4):371-8. 20502238 10.1097/BPO.0b013e3181d8d34d

[7] Vuillermin C Bauer AS Kalish LA Lewine EB Bae DS Waters PM. Follow-up Follow-up Study on the Effects of Tendon Transfers and Open Reduction on Moderate Glenohumeral Joint Deformity in Brachial Plexus Birth Injurytudy on the effects of tendon transfers and open reduction on moderate glenohumeral joint deformity in brachial plexus birth injury. J Bone Jt Surg Am. 2020;102(14):1260-8. 10.2106/JBJS.19.00685 32675676

[8] Waters PM. Smith GR Jaramillo D. Glenohumeral deformity secondary to brachial plexus birth palsy. J Bone Jt Surg Am. 1998;80(5):668-77. 10.2106/00004623-199805000-00007 9611027

[9] Waters PM Bae DS. The effect of derotational humeral osteotomy on global shoulder function in brachial plexus birth palsy. J Bone Jt Surg Am. 2006;88:1035-42. 10.2106/JBJS.E.00680 16651578

[10] Dodwell E O'Callaghan J Anthony A Jellicoe P Shah M Curtis C Clarke H Hopyan S. Combined glenoid anteversion osteotomy and tendon transfers for brachial plexus birth palsy: early outcomes. J Bone Jt Surg Am. 2012;94:2145-52. 10.2106/JBJS.K.01256 23224385

[11] Zargarbashi R Rabie RH Panjavi B Kamran H Mosalamiaghili M Erfani Z Mirghaderi SP Salimi M. Glenoid osteotomy with various tendon transfers for brachial plexus birth palsy: clinical outcomes. J Shoulder Elbow Surg. 2023;32(2):e60-e70. 36115612 10.1016/j.jse.2022.07.026

[12] Dodwell E Hopyan S. Combined glenoid anteversion osteotomy and tendon transfers for brachial plexus birth palsy. JBJS Essent Surg Tech. 2012;2:e23. 31321143 10.2106/JBJS.ST.L.00021 PMC6554082

[13] Le Hanneur M Brahim L Langlais T Bouche PA Fitoussi F. Age influence upon glenohumeral remodeling after shoulder axial rebalancing surgery in brachial plexus birth injury. J Pediatr Orthop. 2023;43:e389-e395. 36882889 10.1097/BPO.0000000000002380

[14] Waters PM Bae DS. The early effects of tendon transfers and open capsulorrhaphy on glenohumeral deformity in brachial plexus birth palsy. Surgical technique. J Bone Jt Surg Am. 2009;91(suppl 2):213-22. 10.2106/JBJS.I.00501 19805585

[15] Pöyhiä T Lamminen A Peltonen J Willamo P Nietosvaara Y. Treatment of shoulder sequelae in brachial plexus birth injury. Acta Orthop. 2011;82(4):482-8. 21657969 10.3109/17453674.2011.588855 PMC3237041

[16] Al-Qattan MM. Total obstetric brachial plexus palsy in children with internal rotation contracture of the shoulder, flexion contracture of the elbow, and poor hand function: improving the cosmetic appearance of the limb with rotation osteotomy of the humerus. Ann Plast Surg. 2010;65:38-42. 20548233 10.1097/SAP.0b013e3181a72f9e

[17] Kirkos JM Papadopoulos IA. Late treatment of brachial plexus palsy secondary to birth injuries: rotational osteotomy of the proximal part of the humerus. J Bone Jt Surg Am. 1998;80:1477-83. 10.2106/00004623-199810000-00009 9801216

[18] McKellar SR Kay J Memon M Simunovic N Kishta W Ayeni OR. Surgical soft tissue management for glenohumeral deformity and contractures in brachial plexus birth injury: a systematic review and meta-analysis. Curr Rev Musculoskelet Med. 2022;15(2):107-20. 35156170 10.1007/s12178-022-09747-6 PMC9076768

[19] Abzug JM Chafetz RS Gaughan JP Ashworth S Kozin SH. Shoulder function after medial approach and derotational humeral osteotomy in patients with brachial plexus birth palsy. J Pediatr Orthop. 2010;30(5):469-74. 20574265 10.1097/BPO.0b013e3181df8604

[20] Abdelgawad AA Pirela-Cruz MA. Humeral rotational osteotomy for shoulder deformity in obstetric brachial plexus palsy: which direction should I rotate? Open Orthop J. 2014;8:130-4. 24987487 10.2174/1874325001408010130 PMC4076617

[21] Assunção JH Ferreira AA Benegas E Bolliger RN Prada FS Malavolta EA Gracitelli ME Camanho GL. Humeral internal rotation osteotomy for the treatment of Erb-Duchenne-type obstetric palsy: clinical and radiographic resultsuchenne-type obstetric palsy: clinical and radiographic results. Clinics (Sao Paulo). 2013;68(7):928-33. 23917655 10.6061/clinics/2013(07)07 PMC3715036

[22] Hultgren T Jönsson K Roos F Järnbert-Pettersson H Hammarberg H. Surgical correction of shoulder rotation deformity in brachial plexus birth palsy: long-term results in 118 patients. Bone Jt J. 2014;96-B(10):1411-8. 10.1302/0301-620X.96B10.33813 25274930

[23] Sarac C Amghar H Nieuwenhuijse MJ Nagels J Buitenhuis SM Wolterbeek R Nelissen R. What range of motion is achieved 5 years after external rotationplasty of the shoulder in infants with an obstetric brachial plexus injury? Clin Orthop Relat Res. 2020;478(1):114-23. 31651590 10.1097/CORR.0000000000000996 PMC7000049

[24] Massamba Vuvu T Dorniol M Le Nen D Thépaut M Brochard S Pons C. Effect of arthroscopic shoulder release on shoulder mobility and bone deformity following brachial plexus birth injury: a systematic review and meta-analysis. J Shoulder Elbow Surg. 2021;30(10):2428-37. 33567353 10.1016/j.jse.2020.12.021

